# Construction of an ECL Detection Platform for Sensitive Detection of Carbaryl Based on an Eu^3+^-Functionalized Metal–Organic Framework Encapsulated with Nanogold

**DOI:** 10.3390/foods11101487

**Published:** 2022-05-19

**Authors:** Chang Liu, Haiyang Wang, Xuelian Hu, Yichuan Cao, Guozhen Fang

**Affiliations:** State Key Laboratory of Food Nutrition and Safety, College of Food Science and Engineering, Tianjin University of Science and Technology, Tianjin 300457, China; liuchang2022666@163.com (C.L.); ocean5416@163.com (H.W.); hxl523686206@126.com (X.H.); cycydx2019@126.com (Y.C.)

**Keywords:** electrochemiluminescence, MOFs, detection, Eu^3+^, carbaryl

## Abstract

In this work, an Eu^3+^-MOF-253@Au electrochemiluminescence sensor was successfully constructed for the first time by encapsulating nanogold in the metal–organic frameworks (MOFs) backbone and pore channels, and assembling Eu^3+^ on the MOF backbone. Firstly, the introduction of nanogold overcomes the weakness of MOFs, which was difficult to achieve, and enhances its catalytic performance, followed by the modification of Eu^3+^ to confer the electrochemiluminescence performance and the function of target detection on the sensor. Moreover, carbaryl was placed in an alkaline working solution to enhance the intensity of electrochemiluminescence signal, as well as to promote the hydrolysis of carbaryl into 1-naphthol, which caused the burst of Eu^3+^-MOF-253@Au electrochemiluminescence sensor, thereby achieving the sensitive detection of carbaryl. On this basis, the electrochemiluminescence detection conditions were optimized, the performance was analyzed, and finally it was successfully used for the detection of carbaryl with good linearity in the range of 0.2–200 μg L^−1^ and a low detection limit (0.14 μg L^−1^).

## 1. Introduction

As an emerging technology in recent decades, electrochemiluminescence (ECL) has been rapidly applied in the analytical fields of environment [1], food [2], clinical [3], and biology studies [4]. Due to the advantages of high sensitivity, high selectivity, fast response, good temporal and spatial control, and simplified setup [5], it has quickly developed into a powerful analysis tool after its discovery around one hundred years ago [6]. Since the first detailed studies in the mid-1960s, the ECL emitter, which is vital in ECL analysis, has been studied and reported on in detail, ranging from the very basic beginning tris(2,2″-bipyridine)ruthenium(II) chloride hexahydrate (Ru(bpy)_3_^2+^) [7] and classic luminol [8], to the booming quantum dots [9], nanocrystals [10], metal nanoclusters [11], and carbon nitride nanosheets [12]. Admittedly, these electrode materials have their own advantages, such as excellent luminescence signals and high sensitivity under certain conditions, but they also have corresponding weaknesses, such as environmental or biological toxicity, as well as poor stability [13]. Therefore, it is of great significance to explore novel electrode materials with remarkably good performance.

A metal–organic framework (MOF) assembled with metal ions and organic ligands under the action of coordination bonds is a crystalline porous material with an adjustable pore structure, a large specific surface area, and open metal sites [14]. In view of its miraculous performance, it has already been successfully applied in the fields of gas adsorption and storage [15], drug transportation [16], and catalysis [17]. On this basis, MOFs with fluorescence characteristics have gradually emerged in the area of detection [18]. However, the organic branches of MOFs are intricate and complicated, which hinders the transfer of electrons in their structure, and makes them poor in terms of electrical conductivity, thereby limiting its application in the electrochemical sensor field. In order to break this barrier, there are two trains of thought: introducing conductive components into the structure of MOFs by inserting metal nanoparticles or carbon-based nanomaterials into the skeleton, and coordinating the functional molecular composition on the skeleton [19]. Alternatively, according to the synthetic tunability and structural regularity of MOFs, electroactive ligands, such as metalloporphyrin and ruthenium complexes, can be used to construct electroactive MOFs [20]. This certainly broadens the application prospects of MOFs significantly.

Carbaryl is a broad-spectrum insecticide with excellent performance, which was discovered in 1956, and was immediately widely used after it was put on the market. At the end of the 1970s, organo-chlorine pesticides were banned, and the usage of pyrethroids, organophosphorus, and carbamate pesticides was increasing year by year. Among them, carbaryl was also widely used as the first carbamate pesticide to be successfully developed. However, a large amount of carbaryl can cause damage to the human body [21], since excessive carbaryl in the environment will enter poultry, livestock, and even the human body through the respiratory tract, skin mucous membrane, or digestive tract, causing damage to the nervous system, muscles, liver, pancreas, and brain as an acetylcholinesterase inhibitor [22]. This means that it is urgent and significant to establish analytical methods for the detection of carbaryl pesticide residues. Therefore, there were also multiple methods for the detection of carbaryl, such as high performance liquid chromatography(HPLC) [23], mass spectrometry(MS) [24], electrochemical method [25], fluorescence method [26], Raman spectroscopy [27], and colorimetry [28]. These detection methods have achieved satisfactory detection results under certain conditions, but there are also some areas that can be improved, such as improving detection efficiency, improving stability, and lowering detection limits.

In this work, nano-gold with conductive and catalytic properties was introduced onto the surface of the MOFs skeleton, which was loaded with luminescent substances, thus establishing an ECL detection platform. Thereinto, europium, a lanthanide metal, has shown luminescence sensitization and antenna effect in fluorescence detection due to its special chemical properties [18]; it has also been found to produce weak emissions at the cathode in ECL detection [29,30]. In addition, when K_2_S_2_O_8_ is used as a co-reactant, ECL can be greatly enhanced [31]. Furthermore, Eu^3+^ was loaded onto the skeleton, and then fixed onto the electrode, which prevented the luminous substance from being dispersed in the working fluid, thereby effectively improving the luminous efficiency and intensity. On the other hand, Eu^3+^ showed a special recognition phenomenon for 1-naphthol, the hydrolysate of carbaryl, in fluorescence detection [32]. Furthermore, the incorporation of nanogold with conductive and catalytic properties into the system can overcome the disadvantage of poor conductivity of MOFs on the one hand, and promote the hydrolysis of carbaryl on the other hand [17]. To go the extra mile, the working solution was adjusted to alkaline to promote the hydrolysis of carbaryl even further, which can also facilitate the occurrence of ECL [33]. These elements of this ECL system complemented each other, and constituted a sensitive and specific electrochemical platform for carbaryl detection.

## 2. Materials and Methods

### 2.1. Reagents

Specific reagents and materials are recorded in the Supplementary Materials.

### 2.2. ECL Measurement

A three-electrode system with a glassy carbon electrode (GCE, 4 mm in diameter) as a working electrode, a platinum disc electrode as a counter electrode, and an Ag/AgCl electrode as a reference electrode was used for the ECL determinations by using an MPI-E II ECL analyzer (Remax Instruments Co., Ltd., Xi’an, China). The electrodes were manufactured by Gaoss Union, Wuhan, China. The ECL measurement was performed in 5 mL of PBS buffer (0.1 M, pH 9) containing 0.05 M K_2_S_2_O_8_ with a scan potential range of 0 V to −1.8 V, a scan rate of 0.2 V s^−1^, and a photomultiplier voltage of 700 V. Furthermore, the cyclic voltammetry (CV) and ECL were recorded simultaneously.

Other instruments and equipment used are recorded in the Supplementary Materials.

### 2.3. Preparation of Eu^3+^-Au@MOF-253 Modified Electrodes

The synthesis of MOF-253 was conducted as reported in previous reports, with some modifications [34,35]: AlCl_3_·6H_2_O (151 mg), glacial acetic acid (859 μL), and 2,2′-bipyridine-5,5′-dicarboxylic acid (153 mg) were added to 9 mL of DMF, and reacted in a reactor at 130 °C for 24 h. Then, the powder was washed three times with DMF, and dried under vacuum conditions at 60 °C for 6 h. The resulting product was washed with methanol for 6 h by Soxhlet extraction, and dried at 60 °C for 6 h under vacuum conditions.

MOF-253@Au were synthesized according to the previous method, with some modifications [36]: MOF-253 (100 mg) was dispersed into 125 mL of anhydrous ethanol containing 3 mL of chloroauric acid solution (25 mmol L^−1^, containing 30 mg HAuCl_4_·3H_2_O). Then, 10 mL of aqueous NaBH_4_ (10 g L^−1^) was added dropwise, and stirred at room temperature for 1 h. The purple product, MOF-253 encapsulated with nanogold, was thus obtained, and washed three times with methanol, then vacuumed overnight.

The synthesized Eu^3+^-MOF-253@Au [37]: MOF-253@Au (100 mg) was dispersed into EuCl_3_·6H_2_O (10 mL, 2 mmol) in anhydrous ethanol, and stirred at room temperature for 24 h. The resulting liquid was then separated by centrifugation, washed three times with anhydrous ethanol to remove the excess Eu^3+^, and finally dried under vacuum conditions at 80 °C for 6 h.

The preparation of Eu^3+^-MOF-253@Au modified electrodes: Eu^3+^-MOF-253@Au (8 mg) was added to 250 μL of anhydrous ethanol and 50 μL of Nafion mixed solution, then was sonicated, and dispersed for 30 min. A 5 μL of mixture was then applied dropwise to the surface of the pretreated GCE and dried at room temperature to obtain the modified Eu^3+^-MOF-253@Au/GCE.

### 2.4. Sample Preparation

Milk and soybean oil were purchased from the local market. Specific steps for sample processing are described in the Supplementary Materials [38,39].

## 3. Results and Discussion

### 3.1. Preparation of Electrodes and Detection of Carbaryl

**Scheme 1:**
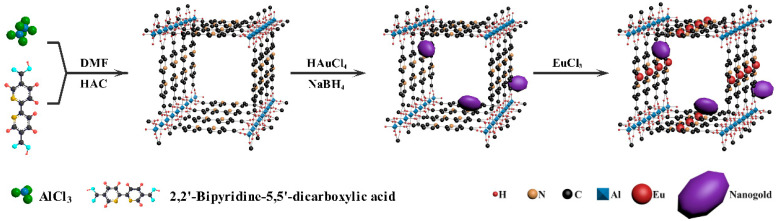
Schematic diagram of the synthesis of Eu^3+^-MOF-253@Au.

As exhibited in Scheme 1, after the synthesis of MOF-253, nanogold was encapsulated on its backbone to enhance its electrical conductivity. Then, Eu^3+^ was immobilized on the open chelation site of MOF-253 as the ECL emitter. In order to analyze whether the strong reducing agent NaBH_4_ had any effect on the structure of MOF-253 when reducing chloroauric acid, scanning electron microscopy (SEM), Fourier transform infrared (FT-IR), and X-ray diffraction (XRD) analyses were performed on MOF-253 and MOF-253@Au, respectively. The patterns are shown in Supplementary Materials.

**Figure 1:**
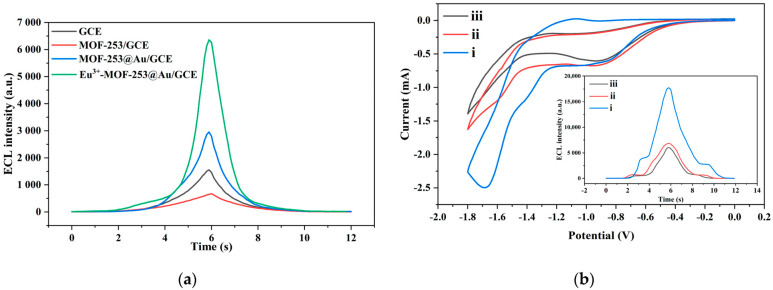
(**a**) ECL curves of different modified electrodes (pH 7); (**b**) CV curves of Eu^3+^-MOF-253@Au/GCE in working solution (i), working solution with 100 μg L^−1^ carbaryl (ii), and 100 μg L^−1^ 1-naphthol (iii); the insert was the corresponding ECL curves (pH 9).

The material was drop-cast onto the surface of GCE for ECL analysis, as shown in Figure 1a. The bare electrode (black) only showed a weak ECL signal, while the MOF-253 modified electrode (red) showed a much weaker ECL, which was related to the fact that the MOF-253 itself was non-conductive. The blue curve showed an enhanced ECL signal for the MOF-253@Au/GCE, which should be attributed to the fact that the conductivity of nanogold encapsulated in MOF-253 increased the electron transfer capability of the electrode surface, thus enhancing the ECL signal. The Eu^3+^-MOF-253@Au/GCE (green curve) showed a significant enhancement of the ECL signal, almost four times that of the bare electrode, and twice that of the MOF-253@Au/GCE, which meant that the prepared Eu^3+^-MOF-253@Au/GCE had a good ECL performance.

**Scheme 2:**
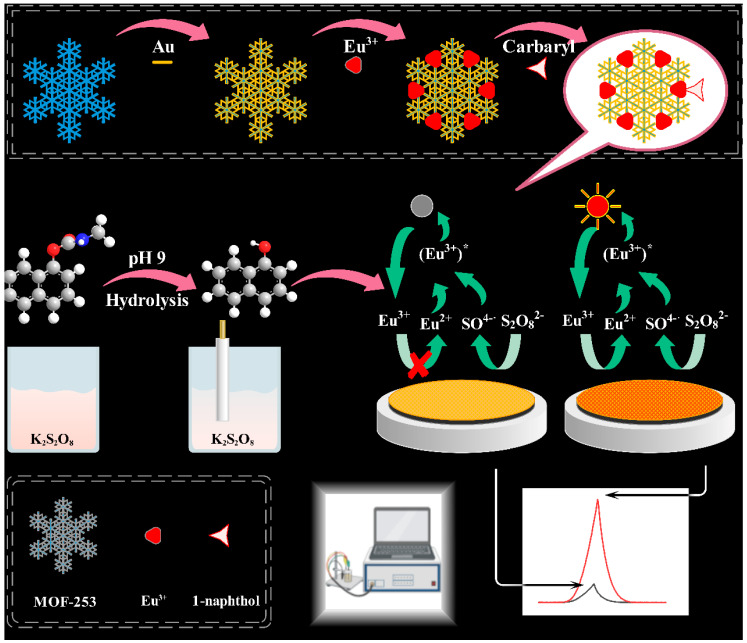
Schematic diagram of the synthesis of Eu^3+^-MOF-253@Au/GCE and carbaryl detection.

The principle of ECL detection of carbaryl was speculated as follows, according to previous literature reports: when the initial negative potential was applied to the electrode surface, the Eu^3+^ fixed on the electrode surface was charged and reduced to Eu^2+^. Meanwhile, the co-reactant S_2_O_8_^2^^−^ in the solution diffused to the electrode surface, and was reduced to strong oxidizing anions SO_4_^−^^•^ and SO_4_^2^^−^. Subsequently, the SO_4_^−^^•^ reacted with Eu^2+^, and transferred energy to Eu^2+^ to generate an excited state (Eu^3+^)*; then (Eu^3+^)* returned to the ground state to release photons. This process is illustrated in Equations (1)–(4), where *hv* represents the photon released when the excited state returns to the ground state. However, carbaryl was added to the working solution. and it decomposed rapidly to 1-naphthol in alkaline solution due to the electric, as well as the catalytic effect of nanogold [40,41]. The specific recognition of 1-naphthol by Eu^3+^-MOF-253@Au was speculated to have several possibilities: first, the 1-naphthol obtained from the hydrolysis of carbaryl was more active and more amenable to electrochemical reactions due to the presence of the naphthalene ring [42]; second, an electron transfer between Eu^3+^-MOF-253@Au and 1-naphthol [43]; third, an energy transfer from the excited state of (Eu^3+^)* was transferred to 1-naphthol or the oxide it formed [44,45]; fourth, electrostatic interactions between the free Lewis base site of the pyridine ring in Eu^3+^-MOF-253@Au and the hydroxyl group of 1-naphthol [32,46]; and fifth, the possible formation of a complex between 1-naphthol and Eu^3+^ that prevents the reduction of exposed Eu^3+^ in the material to Eu^2+^. This leads to the bursting of the ECL. The above process can be represented by Scheme 2. Meanwhile, this conjecture was verified using cyclic voltammetry, and the result is presented in Figure 1b. Curves (i), (ii), and (iii) were the CV curves of the Eu^3+^-MOF-253@Au/GCE in a working solution with 100 μg L^−1^ carbaryl, and with 100 μg L^−1^ 1-naphthol, respectively. It can be seen that the original obvious redox peak became significantly smaller after the addition of the target, probably because the hydroxyl group of naphthol complexed with Eu^3+^, which blocked the pathway of Eu^3+^ participation in the ECL process.

Eu^3+^+e^−^→Eu^2+^ (1)

S_2_O_8_^2−^+e^−^→SO_4_^−•^+SO_4_^2−^ (2)

Eu^2+^+SO_4_^−•^→(Eu^3+^)*+SO_4_^2−^ (3)

(Eu^3+^)* →Eu^3+^+*hv* (4)

### 3.2. Optimization of ECL Detection Platform

**Figure 2:**
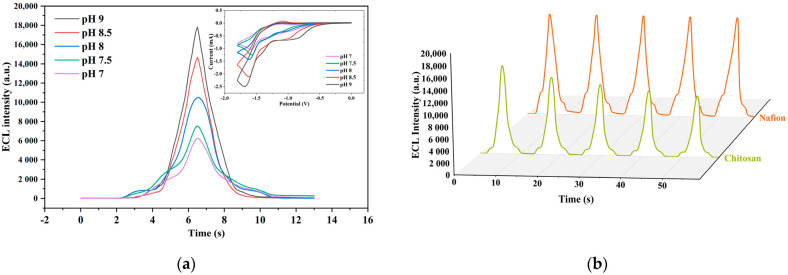
(**a**) Optimization of pH value of working solution, insert was CV curves at different pH values; (**b**) ECL curves of electrodes modified with Nafion and chitosan, respectively.

There were various factors affecting the detection results, but the detection principle of this method was based on the rapid hydrolysis of carbaryl in solution; as such, the selection of the pH value of the detection solution was crucial. Additionally, since pH also affects the strength of the ECL signal, a high pH might favor the formation of the (Eu^3+^)* [33]. Taking the above into consideration, the pH of the detection solution was adjusted for detection from 7, gradually increasing in order to achieve the optimal level of ECL signal for the constructed sensor, taking into account the practical operation, the electrode working environment, and the stability of the synthesized material. The results are shown in Figure 2a. The ECL signal gradually increased during the increase in pH from 7 to 9, and the intensity of the ECL signal had reached an appreciable 17,825 a.u. when the pH reached 9. The corresponding CV curves in Figure 2a show that the peak current increased with increasing pH, which may be because the increase in pH contributed to the redox reaction in the system, which, in turn, increased the electron transfer and generated more excited states (Eu^3+^)*, thereby increasing the ECL signal intensity. At the same time, the ECL quenching values of carbaryl and 1-naphthol to the sensor were compared and analyzed at different pH values. The results showed that when the pH value reached 9, the quenching values of carbaryl were consistent with that of 1-naphthol at the corresponding concentration Therefore, the pH of the working solution was adjusted to 9 in the subsequent measurements.

In order to immobilize the synthesized material on the electrode surface without affecting the detection of the electrode, both Nafion and chitosan solutions (1%), as common electrode modifiers in electrochemical detection, were used to immobilize Eu^3+^-MOF-253@Au onto the GCE surface to prepare the corresponding electrodes. As shown in Figure 2b, the ECL signal of the chitosan-modified electrode was weaker, and gradually decreased compared with that of the Nafion-fixed electrode, probably because the material was shed during the detection process, indicating that chitosan was not suitable for the fixation of the electrode modification material prepared in this study. On the contrary, the ECL signal of the electrode modified by the Nafion solution was more stable and remained above 17,000 a.u; therefore, the Nafion solution was used for the immobilization of the electrode material in the subsequent experiments.

### 3.3. Performance of ECL Detection Platform

**Figure 3:**
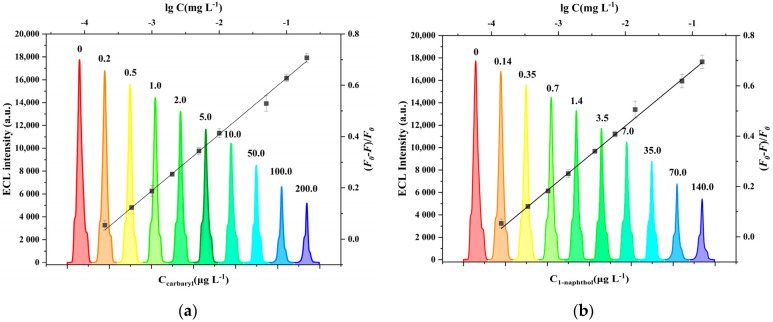
(**a**) ECL curves of a series concentration of carbaryl (0.2, 0.5, 1.0, 2.0, 5.0, 10.0, 50.0, 100.0, and 200.0 μg L^−1^) detected by Eu^3+^-MOF-253@Au sensor, and the calibration curve for carbaryl detection; (**b**) ECL curves of a series concentration of 1-naphthol (0.14, 0.35, 0.7, 1.4, 3.5, 7.0, 35.0, 70.0, and 140.0 μg L^−1^) detected by Eu^3+^-MOF-253@Au sensor, and the calibration curve for 1-naphthol detection.

The electrode was modified and assayed according to the conditions obtained from the above optimization experiments to evaluate its recognition, as well as the detection ability for the targets. Figure 3a shows that the ECL signal intensity of the prepared electrode gradually decreased with increasing carbaryl concentration in the range of 0.2–200 μg L^−1^, and the logarithm of the carbaryl concentration (mg L^−1^) showed a good linear relationship with the relative change value of ECL signal intensity (*F*_0_ − *F*)/*F*_0_ of the Eu^3+^-MOF-253@Au/GCE. Where *F*_0_ represents the initial fluorescence intensity of the Eu^3+^-MOF-253@Au/GCE in the working solution, and *F* represents the fluorescence intensity obtained by the Eu^3+^-MOF-253@Au/GCE detecting a certain concentration of the target solution. The linear regression equation was as follows: $\left(F_{0}-F\right)/F_{0}=0.21956\mathrm{lgC}+0.84778$ (R^2^ = 0.9988), and the detection limit was calculated as 0.14 μg L^−1^.

In order to verify whether the ECL signal variation was due to 1-naphthol obtained by the hydrolysis of carbaryl, 1-naphthol solutions with corresponding concentrations were prepared and measured under the same conditions (based on the molar mass calculation, hydrolysis of 1 mg carbaryl yields around 0.7165 mg of 1-naphthol; therefore, the solutions of 0.07, 0.14, 0.35, 0.7, 1.4, 3.5, 7.0, 35.0, and 70.0 μg L^−1^ of 1-naphthol were configured). The curves in Figure 3b show the variation of the ECL signal intensity when the electrode measured 0.14–140 µg L^−1^ of 1-naphthol solution, as well as the calibration curve plotted from the signal variation calculation. The regression curve equation for 1-naphthol was calculated as follows: $\left(F_{0}-F\right)/F_{0}=0.21996\mathrm{lgC}+0.87963$ (R^2^ = 0.9983). The change of the ECL curve in Figure 3b essentially matches the trend of the change of the ECL curve in Figure 3a, and the regression equation of the fitted curve in Figure 3b also almost agrees with the slope of the equation obtained from the detection of carbaryl, which means that it can essentially be concluded that 1-naphthol can lead to the change of the ECL signal intensity of the Eu^3+^-MOF-253@Au/GCE.

### 3.4. Selectivity, Precision and Stability

**Figure 4:**
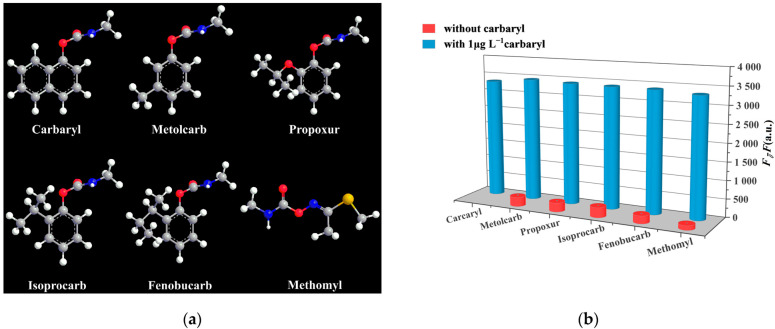
(**a**) The chemical structure of carbaryl and its structural analogs; (**b**) the selectivity of the Eu^3+^-MOF-253@Au/GCE towards carbaryl and interferents.

The ability to recognize the target is one of the important properties of the sensor, so several carbamate pesticides (metolcarb, propoxur, isoprocarb, fenobucarb, methomyl) were selected as interferents to evaluate the selectivity of the prepared electrodes. The interferents’ chemical structure formulae are shown in Figure 4a. Figure 4b shows that the ECL quenching value (*F*_0_ − *F*) of the prepared Eu^3+^-MOF-253@Au/GCE for carbaryl (1 μg L^−1^) was several times higher than that for other analytes of the same concentration. The ECL quenching values (*F*_0_ − *F*) of the sensor increased more in the presence of interferents than in the absence of interferents, which may be due to the effect of the similar structure of the interferents. The quenched values (*F*_0_ − *F*) with and without the interferents were analyzed by analysis of variance (ANOVA), and the *F*-test showed no significant difference in the values of ECL signal changes in the presence and absence of the interferents. It was probably because they and their hydrolysis products were not able to complex with Eu^3+^ chelated on MOF-253, indicating the good selectivity and anti-interference performance of the prepared sensor.

**Figure 5:**
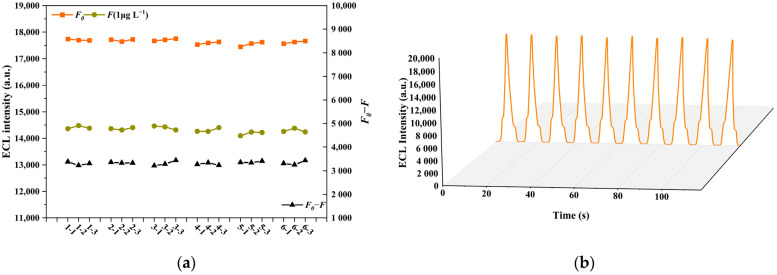
(**a**) The precision of the Eu^3+^-Au@MOF-253 sensor; (**b**) the ECL curve for 10 consecutive detections carried out by Eu^3+^-MOF-253@Au/GCE.

To evaluate the precision of the constructed sensors, six electrodes were prepared using the same modification method for parallel experiments. The initial signal intensity *F*_0_, the ECL signal intensity *F* for 1 μg L^−1^ carbaryl, and the difference (*F*_0_ − *F*) were determined and analyzed separately. It can be seen from Figure 5a that the prepared Eu^3+^-MOF-253@Au sensor has a stable ECL signal. The ANOVA performed on the (*F*_0_ − *F*) values revealed that there was no significant difference in the detection of carbaryl by the six electrodes, and that the prepared sensor has reliable reproducibility (The *F*-test results were: *F* = 0.4932 < 3.106 = *F*(6–1, 18–6)), α = 0.05.

Stability is also an important index of electrochemical sensors, so the same electrode was measured 10 times continuously, and its signal change was analyzed to assess the stability of the Eu^3+^-MOF-253@Au sensor. In addition, the prepared sensor, after measuring its ECL signal, was stored at 4 °C, and its signal intensity was measured every 6 days to evaluate the stability after long-term storage in three groups operated in parallel. The results are shown in Figure 5b and Figure S3, respectively. The Eu^3+^-MOF-253@Au sensor displayed excellent stability for 10 consecutive measurements (Relative Standard Deviation, RSD = 0.65%) and long-term storage (RSD = 1.32%). The low RSD indicated a good reproducibility and high precision of the sensor. Moreover, a T-test was performed on the same Eu^3+^-MOF-253@Au/GCE for inter-day and intra-day measurements of carbaryl at 1 μg L^−1^. The results showed no significant difference between the two datasets. This result again demonstrates the accuracy of the established assay.

### 3.5. Real Sample Analysis

According to the regulations, the EU’s maximum residue levels (MRL) of carbaryl residue in milk is 0.05 mg kg^−1^ [47], which is the same as China’s, while the EU’s carbaryl residue in soybeans and China’s carbaryl residue in soybean oil is 0.05 mg kg^−1^ and 1 mg kg^−1^, respectively [48]. Milk and soybean oil were used as actual samples for spike recovery experiments, and the ECL determination results were verified by HPLC simultaneously. The results of the analysis and calculation are presented in Table 1, and the recoveries were calculated to be 76.5–95.4% for three different concentration levels spiked on two actual samples. A paired sample T-test between spiked and detected concentrations showed *p* = 0.07174 > 0.05, indicating that there was no significant difference between the spiked and detected concentrations. This result proved that the constructed Eu^3+^-Au@MOF-253 sensor can be applied to the analysis of real samples.

### 3.6. Comparison of Different Detection Methods

The performance of the trace carbaryl detection strategy based on the constructed Eu^3+^-MOF-253@Au sensor was compared with previously reported carbaryl detection methods, the result of which is presented in Table 2. It is clear that the established ECL method can balance high detection efficiency, large detection range, and low detection limit with excellent performance. It also showed lower limit of detection (LOD), as well as good recoveries and low RSDs in food samples compared to other ECL methods that selected water as the actual sample. This was attributed to the following aspects: (1) the excellent thermal and chemical stability of the MOFs material laid the structural foundation for the construction, modification, and long-term storage of the sensor; (2) the excellent chemical and fluorescence properties of Eu^3+^ facilitated the recognition of 1-naphthol by Eu^3+^-MOF-253, thereby avoiding the superposition of multiple recognition elements and providing good selectivity; (3) the nanogold dispersed on the backbone of the MOFs had good electrical conductivity, which improved the electrode surface electron transfer rate, and effectively amplified the ECL response; (4) placing the target carbaryl in the detection solution adjusted to alkaline was beneficial for the rapid completion of its decomposition reaction and uniform contact with the electrode, which also improved the detection efficiency. On the other hand, it was presumed that the alkaline detection solution was conducive to the formation of the excited state of the luminol, effectively enhancing the ECL intensity.

## 4. Conclusions

In this study, based on the selectivity of Eu^3+^-MOF-253 for 1-naphthol, nanogold was introduced into the MOF framework to enhance the catalytic properties and conductivity of Eu^3+^-MOF-253 to construct an ECL sensor for the sensitive detection of carbaryl. After a series of optimization and evaluation, it was found that the established assay showed admirable detection efficiency, sensitivity, low detection limit (LOD = 0.14 μg L^−^^1^), and high stability, as well as demonstrating satisfactory recoveries in actual samples (76.5–95.4%), which implies good application prospects. This work not only proposes a rapid and sensitive method for the detection of carbaryl residues in foods, but also enriched the method for the determination of hazard factors in food, and expanded the application of MOFs in the field of electrochemistry.

## Figures and Tables

**Table 1:** Determination of carbaryl in food samples by the proposed ECL and HPLC.

Sample	Added (μg kg^−1^ /μg L^−1^)	HPLC		ECL	
		Found (μg kg^−1^ /μg L^−1^)	Recovery (Mean ± SD, *n* = 3)	Found (μg kg^−1^ /μg L^−1^)	Recovery (Mean ± SD, *n* = 3)
Milk	0	not detected	—	not detected	—
	1	0.87	87.1 ± 1.5	0.82	82.4 ± 2.4
	50	47.42	94.8 ± 3.4	47.57	95.1 ± 3.6
	100	97.73	97.7 ± 1.9	95.42	95.4 ± 2.2
Soybean oil	0	not detected	—	not detected	—
	1	0.83	82.7 ± 2.6	0.77	76.5 ± 2.5
	50	44.12	89.1 ± 2.6	41.53	83.1 ± 3.1
	100	93.51	93.5 ± 1.8	86.33	86.3 ± 1.7

n: the number of measurements.

**Table 2:** Comparison of the proposed method with other reported methods in the literature.

Method	Detection Range	LOD	Sample	Recovery (%)	RSD (%)	Year	References
High performance liquid chromatography-diode array	1.0 to 320.0 ng mL^−1^	0.12–0.40 ng mL^−1^	Milk, White wine	86.5–99.2	3.2–5.7	2019	[23]
	0.5 to 160.0 ng mL^−1^	0.06–0.20 ng mL^−1^	Juice	82.0–104.7	2.8–4.5		
Electrospray ionization mass spectrometry	20.00–1000.00 ng mL^−1^	1.16–4.18 ng g^−1^	Honey	87.00–114.98	0.97–6.22	2017	[24]
DNA aptamer	100–1500 nmol L^−1^	15.23 nmol L^−1^	Tap water, Xuanwu Hu	97.7–107.3%	0.8–2.5	2021	[26]
Surface-enhanced Raman scattering	10^−4^ − 10^−9^ g mL^−1^ (situ detection)	0.77 ng mL^−1^ (situ detection)	Cherry	--	--	2021	[27]
	10^−3^ − 10^−8^ g mL^−1^ (swabbing detection)	5.8 ng mL^−1^ (swabbing detection)		--	--		
Colorimetric	10 μM-5 mM	10 ppm	Apple	--	--	2018	[28]
ECL	0–18 μM	1.0 nM	--	96.9–101.7	1.87–3.02	2013	[49]
ECL	1.0 × 10^−^^12^–1.0 × 10^−7^ M	1.0 × 10^−12^ M	Tap water	94.0–95.8%	3.53–4.62	2020	[50]
ECL	5 × 10^−4^–10 mg mL^−1^	2 × 10^−4^ μg mL^−1^	River water	99.0–108.0	5%<	2015	[51]
ECL	0.2–200 μg L^−1^	0.14 μg L^−1^	Milk, Soybean oil	76.5–95.4%	2.0–3.8	2021	This work

## Data Availability

The data presented in this study are available on request from the corresponding author.

## Supplementary Materials

The following supporting information can be downloaded at: https://www.mdpi.com/article/10.3390/foods11101487/s1, Reagents and Materials, Apparatus and Instruments, Sample preparation, Characterization of MOF-253, MOF-253@Au and Eu^3+^-MOF-253@Au, Calculation of detection limit. Figure S1: The SEM image of MOF-253 (A) and MOF-253@Au (B); Figure S2: (A) The FT-IR patterns of MOF-253 and MOF-253@Au. (B) The XRD patterns of MOF-253 and MOF-253@Au. (C) The X-ray photoelectron spectra of MOF-253, MOF-253@Au, and Eu-MOF-253@Au. (D) The N2 sorption isotherm at 77K for MOF-253 and Eu-MOF-253@Au; Figure S3: The variation for ECL responses of proposed sensor after long-term storage.
