# Supplementary material for: Construction of an ECL Detection Platform for Sensitive Detection of Carbaryl Based on an Eu3+-Functionalized Metal–Organic Framework Encapsulated with Nanogold
Source: Foods. 2022 May 19;11(10):1487. doi: 10.3390/foods11101487 (PMC9141832; doi:10.3390/foods11101487)
Supplement: Supplementary file 1 [file foods-11-01487-s001.zip › foods-1691326-supplementary.pdf]

Construction of ECL detection platform for sensitive detection of carbaryl based on an Eu^{3+}-functionalized metal–organic framework encapsulated with nanogold

Chang Liu [a], Haiyang Wang [a], Xuelian Hu [a], Yichuan Cao [a], Guozhen Fang [a,*]

[a] State Key Laboratory of Food Nutrition and Safety, Tianjin University of Science and Technology, Tianjin 300457, China

Corresponding Authors：

* fangguozhen@tust.edu.cn (Guozhen Fang),

Tel: (+86 22) 60912493

Fax: (+86 22) 60912493

Reagents and Materials

Carbaryl (99.9%), metolcarb (99.7%), propoxur (99.9%), fenobucarb (98.4%), methomyl (99.9%) and isoprocarb (97.6%) were purchased from Dr. Ehrenstorfer GmbH (Germany). Glacial acetic acid (HAC), N, N-Dimethylformamide (DMF) were supplied by Sinopharm Chemical Reagent Co.. Europium Chloride ($EuCl_3$，99.9%) and 2,2'-Bipyridine-5,5'-dicarboxylic acid (98%+) were achieved from Acros Organics (USA) and Adamas-beta Reagent Co. (Shanghai, China), respectively. Nafion’D520 dispersion (5%, w/w in water and 1-propanol) and Aluminum chloride hexahydrate ($AlCl_3{\cdot}6H_2O$, 99%) were obtained from Alfa Aesar (Shanghai, China). PBS buffer (10×0.01M, pH7.2-7.4) and potassium peroxodisulfate ($K_2S_2O_8$) were purchased from Solarbio (Beijing, China) and Sigma-Aldrich (Shanghai, China). Methanol, ethanol,

potassium hydroxide (KOH), sodium borohydride ($NaBH_4$) were obtained from Tianjin Chemical Reagent Plant (Tianjin, China). All of the reagents were at least analytical grade. Doubly deionized water (DDW, 18.2 MΩ cm^{-1}) obtained from a Milli-Q water purification system was used throughout the experimental.

Apparatus and Instruments

Scanning electron microscopy (SEM, S4800, HITACHI, Tokyo, Japan), Fourier Transform Infrared Spectroscopy (FT-IR, spectrum 100, Perkin Elmer Inc, USA), X-ray diffraction (XRD, D8advance, Buker, Germany) and BET (Auto-sorb-iQA3200-4, Quantatech Co., USA) were used to observe the surface morphological characteristics and structural features of the MOF-253 and Au@MOF-253, respectively. And the X-ray photoelectron spectra (XPS, 250xi, Thermo ESCALAB, USA) was used for elemental analysis of MOF-253, Au@MOF-253, and Eu-Au@MOF-253.

Sample preparation

Milk[1]: 5.00 g of the sample was placed in a 50 mL centrifuge tube, to which a small amount of carbaryl solution was added to make the concentration of carbaryl in the sample equal to 1 μg kg^{-1}, 50 μg kg^{-1}, and 100 μg kg^{-1}, respectively, and left overnight in the dark in a refrigerator to allow the carbaryl to fully penetrate into the sample. 20 mL of acetonitrile, 5 g of anhydrous sodium sulfate and 2 g of sodium chloride were added to the samples overnight and homogenized for 1 min in order, followed by centrifugation at 5000 r min^{-1} for 5 min, and the supernatant was aspirated. The residue obtained by centrifugation was extracted once again with 10 mL acetonitrile, and the supernatant was combined and concentrated to near dryness by rotary evaporation in a water bath at 40 °C, dissolved with 5 mL methanol, and left to purify.

After activation of the C_{18} solid phase extraction column with 10 mL of methanol, the extract was sampled, and then eluted with methanol (flow rate not exceeding 1 mL

min^{-1}). The entire eluate was collected in a flask and concentrated to near dryness by rotary evaporation in a water bath at 40 ℃. Then it was blown dry with high purity nitrogen, and dissolved with 0.5 mL of methanol solution, filtered through a 0.22 μm filter membrane and stored in a refrigerator at 4 °C for determination.

Soybean oil[2]: 3.00 g of the sample was weighed in a 50 mL plastic centrifuge tube, to which a small amount of carbaryl solution was added to make the concentration of carbaryl in the sample equal to 10 μg kg^{-1}, 500 μg kg^{-1}, 1000 μg kg^{-1}, and placed in the dark in the refrigerator overnight to make the carbaryl fully penetrate into the sample. Then, 5 mL of water, 15 mL of acetonitrile, 6 g of anhydrous magnesium sulfate, 1.5 g of sodium acetate and 1 ceramic homogenizer were added into the overnight samples sequentially, shaken vigorously for 1 min, and centrifuged at 4500 r min^{-1} for 5 min.

Eight milliliters of the supernatant were aspirated accurately and added to a 15 mL plastic centrifuge tube containing 1200 mg of anhydrous magnesium sulfate, 400 mg of PSA (ethylenediamine-N-propylsilane silica gel, 40-60 μm) and 400 mg of C_{18} (octadecylmethylsilane modified silica gel, 40-60 μm), vortexed for 2 min, and then centrifuged at 4200 r min^{-1} for 5 min. 5 mL of supernatant was aspirated in a 10 mL centrifuge tube, purged dry with nitrogen in a water bath at 50 °C. Finally, 0.75 mL of methanol was added accurately, vortexed and filtered through a 0.22 μm microporous membrane, and stored at 4°C for measurement.

Characterization

As shown in **Figure S1.**, MOF-253 was a clustered petal-shaped structure. After the reduction of $HAuCl_4$ by $NaBH_4$, the MOF-253 became more coarse and dispersed, but it still maintains its three-dimensional independent structure, which also indicated the good stability of MOF-253.

The FT-IR patterns in **Figure S2(A)**. also showed that MOF-253 and MOF-253@Au have the same absorption peaks, which meant that the chemical bonds of

MOF-253 were not broken before and after the nanogold modification. The XRD patterns in **Figure S2(B)**. reconfirmed the consistency of the structure of MOF-253 with MOF-253@Au. Also, these data obtained from the above characterization were the same as those in the relevant literature[3].

After the introduction of Eu^{3+}, X-ray photoelectron spectroscopy (XPS) was performed to verify the modification results, and the obtained results are shown in **Figure S2(C)**. The energy spectrum curve a of MOF-253 showed the peaks of O (535 eV), N (399 eV), C (285 eV), and Al (74 eV), respectively, and the curve b of MOF-253@Au added the peak of Au (84 eV), and the peak of Eu (1136 eV) was added to the energy spectrum curve c of Eu^{3+}-MOF-253@Au. which indicates that the modification of the material is successful. In addition, the BET curves in **Figure S2(D)** corroborate the successful modification of Eu^{3+} and Au onto MOF-253.

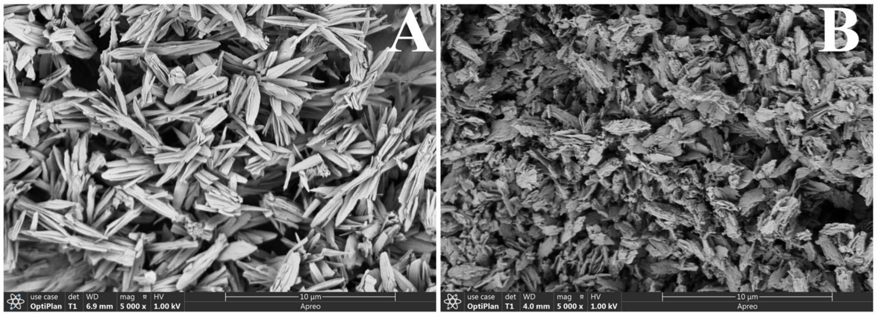

Figure S1. The SEM image of MOF-253(A) and MOF-253@Au(B).

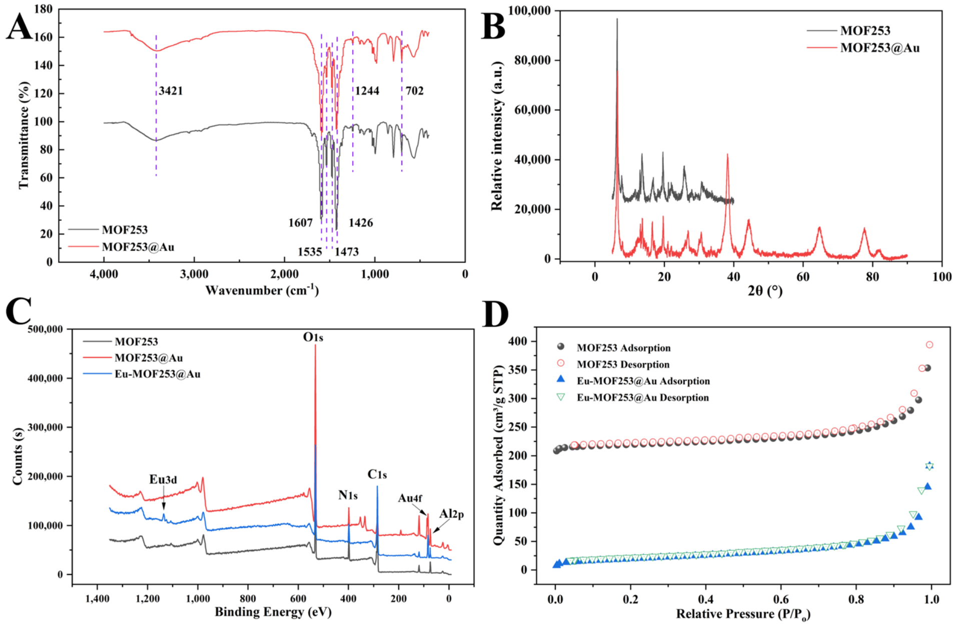

Figure S2. (A)The FT-IR patterns of MOF-253 and MOF-253@Au. (B)The XRD patterns of MOF-253 and MOF-253@Au. (C)The X-ray photoelectron spectra of MOF-253, MOF-253@Au, and Eu-MOF-253@Au. (D)The N_2 sorption isotherm at 77K for MOF-253 and Eu-MOF-253@Au.

Reproducibility and stability

The ECL signal of the sensors were detected every six days, five times in total in three parallel, and the results were shown in **Figure S3,** which indicated that the Eu^{3+}-MOF-253@Au sensor maintained a strong ECL signal intensity (RSD = 1.32%) despite the weakening of the Eu^{3+}-MOF-253@Au sensor during long-term storage.

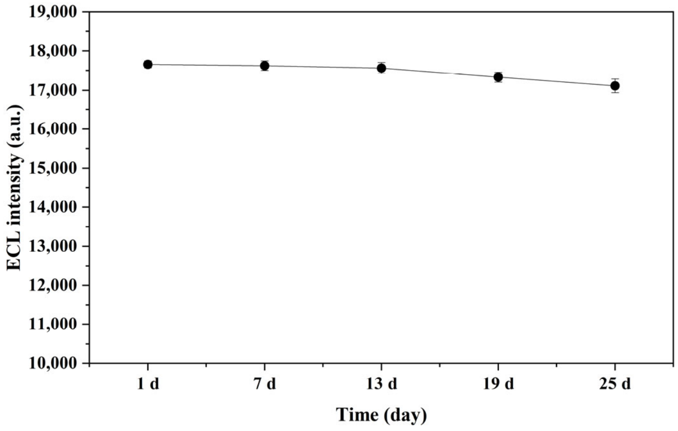

Figure S3. The variation for ECL responses of proposed sensor after long-term storage.

Calculation of detection limit

Referring to the U.S. Environmental Protection Agency's method, repeat the spiked sample at least seven times at low concentrations, where the spiked concentration is generally 1-5 times the expected method detection limit, and then calculate the method detection limit (MDL) according to the formula below:

$$MDL = S * t(n-1, 1-a = 0.99)$$

Where n is the number of samples repeatedly measured, S is the standard deviation of n measurements, t is the t-value of Student's t test when the degree of freedom is (n-1), and 1-a is the confidence level.

This method repeatedly measures the changes of ECL response values of 0.15 μg L^{-1} carbaryl solution 10 times to calculate the detection limit.

References:

1. China, S. F. a. D. A. o. t. P. s. R. o., National food safety standards-Determination of multiple carbamate pesticides residues in milk and dairy products Liquid chromatography-mass spectrometr. In *GB 23200.90-2016*, 2016; p 2.
2. China, S. A. o. M. S. a. A. o. t. P. s. R. o., National food safety standards-Determination of 9 carbamate pesticides and metabolites residues in foods of plant origin-Liquid chromatography-post-colum derivatization. In *GB 23200.112-2018*, 2018; p 3.
3. Bloch, E. D.; Britt, R.; Lee, R.; Doonan, R. J.; Uribe-Romo, R. J.; Furukawa, H.; Long, R. R.; Yaghi, R. M., Metal insertion in a microporous metal-organic framework lined with 2,2'-bipyridine. *Journal of the American Chemical Society* **2010,** *132* (41), 14382. https://doi.org/10.1021/ja106935d
